# Systematic Identification of Hub Genes in Placenta Accreta Spectrum Based on Integrated Transcriptomic and Proteomic Analysis

**DOI:** 10.3389/fgene.2020.551495

**Published:** 2020-09-15

**Authors:** Bingnan Chen, Di Wang, Yue Bian, Jiapo Li, Tian Yang, Na Li, Chong Qiao

**Affiliations:** ^1^Department of Obstetrics and Gynecology, Shengjing Hospital of China Medical University, Shenyang, China; ^2^Key Laboratory of Maternal-Fetal Medicine of Liaoning Province, China Medical University, Shenyang, China; ^3^Key Laboratory of Obstetrics and Gynecology of Higher Education of Liaoning Province, Shenyang, China; ^4^Research Center of China Medical University Birth Cohort, Shenyang, China; ^5^Department of Internal Medicine, Shengjing Hospital of China Medical University, Shenyang, China

**Keywords:** placenta accreta spectrum, biomarker, transcriptomics, proteomics, integrated analysis, bioinformatics

## Abstract

Placenta accreta spectrum (PAS) is a pathological condition of the placenta with abnormal adhesion or invasion of the placental villi to the uterine wall, which is associated with a variety of adverse maternal and fetal outcomes. Although some PAS-related molecules have been reported, the underlying regulatory mechanism is still unclear. Compared with the study of single gene or pathway, omics study, using advanced sequencing technology and bioinformatics methods, can increase our systematic understanding of diseases. In this study, placenta tissues from 5 patients with PAS and 5 healthy pregnant women were collected for transcriptomic and proteomic sequencing and integrated analysis. A total of 728 messenger RNAs and 439 proteins were found to be significantly different between PAS group and non-PAS group, in which 23 hub genes were differentially expressed in both transcriptome and proteome. Functional enrichment analysis showed that the differentially expressed genes were mainly related to cell proliferation, migration and vascular development. Totally 18 long non-coding RNA were found that might regulate the expression of hub genes. Many kinds of single nucleotide polymorphism, alternative splicing and gene fusion of hub genes were detected. This is the first time to systematically explore the hub genes and gene structure variations of PAS through integrated omics analysis, which provided a genetic basis for further in-depth study on the underlying regulatory mechanism of PAS.

## Introduction

Placenta accreta spectrum (PAS) is a pathological condition of the placenta with abnormal adhesion or invasion of the placental villi to the uterine wall (Society of Gynecologic Oncology et al., 2018). There are three types of PAS, including placenta accreta, placenta increta and placenta percreta (Jauniaux et al., 2018b). In recent years, the prevalence of PAS has increased, which may be directly related to the increase in cesarean section rates in most high-income and middle-income countries (Thurn et al., 2016; Jauniaux et al., 2018a). The prevalence of PAS has increased about eight times since 1970s (Silver and Branch, 2018). A study showed that the overall proportion of PAS in recent years had even reached 0.91% (El Gelany et al., 2019).

Placenta accreta spectrum is associated with a variety of adverse maternal and fetal outcomes, including preterm birth, low birth weight infants, increased perinatal mortality, maternal rupture of the uterus and postpartum hemorrhage, etc. (Kabiri et al., 2014; Vinograd et al., 2015; Farquhar et al., 2017). This makes PAS become one of the important factors that affect the safety and prognosis of mothers and fetuses in perinatal period. The pathogenesis of PAS is mainly attributed to the absence of decidual or basal layer, abnormal maternal vascular remodeling and excessive invasion of extravillous trophoblasts (EVTs) (Tantbirojn et al., 2008). However, the hub genes and underlying mechanism involved in PAS are still poorly understood.

With the development of the next-generation sequencing technology, the bioinformatics analysis has enabled us to understand the full picture of the biological sample under a disease on a multi-omics level (Bani Baker and Nuser, 2019). Because of the presence of post-translational control, the true state of cells or tissues can not be reflected accurately only through transcriptomics research. With the development of proteomics technology, the integrated transcriptomic and proteomic analysis has become a powerful tool to discover the regulation of gene expression (Vogel and Marcotte, 2012). The application of bioinformatics methods allowed many hub genes to be found as potential therapeutic targets in cancer research (Blum et al., 2018; Danaher et al., 2018; Poma et al., 2018). However, there are still few bioinformatics studies on PAS. In this study, transcriptome and proteome sequencing was performed in the placenta tissues of 5 women with PAS and 5 healthy pregnant women. Through the integrated analysis, the hub genes closely related to PAS were screened out, and the gene structure was preliminarily explored. The results provided evidence for the further study on the underlying regulatory mechanism of PAS.

## Materials and Methods

### Study Population

Patients diagnosed with PAS and healthy pregnant women of maternal age and gestational age at delivery matched in a large teaching hospital of north China were included in this study. Studies have shown that most patients with PAS have placenta previa and/or prior cesarean section (Thurn et al., 2016; Jauniaux et al., 2018a). Considering the interference of these factors to the study, the inclusion criteria for this study were as follows: placenta previa, prior cesarean section, cesarean delivery this time, singleton. And the exclusion criteria were: other uterine cavity operation or uterus-related diseases (such as uterus bicornis and adenomyosis), obstetric complications (such as hypertensive disorder complicating pregnancy, gestational diabetes), systemic disease. Patients with preterm birth were not excluded from this study because patients with PAS are at significantly increased risk of preterm birth (Vinograd et al., 2015). The diagnosis of PAS was based on the International Federation of Gynecology and Obstetrics (FIGO). According to the clinical and histologic criteria in FIGO, PAS was divided into placenta creta, increta and precreta (Jauniaux et al., 2019). Placenta previa was diagnosed by ultrasound and further divided into complete placenta previa (placenta completely covered the cervical internal os) and incomplete placenta previa (placenta margin reached or partially covered the cervical internal os) (Orbach-Zinger et al., 2018). Diagnosis of other diseases was based on the International Classification of Diseases, 11th edition (ICD-11)^1^. The study was approved by the Medical Ethics Committee of China Medical University. All participants signed written informed consent.

### Collection of Placenta Tissues

A piece of placenta tissue on maternal side for each patient was taken immediately after cesarean section. The placental tissues of patients with PAS were taken at the place of accreta, containing villi, decidua and myometrial fibers. And the placental tissues of patients without PAS were taken at the place which was 1/2 length of the placenta radius from the umbilical cord. Since no PAS cases required hysterectomy in this study, the placenta tissue that deeply invaded the uterus was not obtained. The collected placenta tissues were rinsed with sterile saline. Tissues were then frozen in liquid nitrogen and stored at −80°C until processing transcriptomic and proteomic analysis.

### RNA-Seq

Total RNA was extracted from placenta samples using Trizol (Invitrogen, Carlsbad, CA, United States) according to the manual instructions. For placenta samples, grind about 60 mg with liquid nitrogen into powder and transfer the powder samples into the 2 ml tube contains 1.5 ml Trizol reagent. The mix was centrifuged at 12000 × *g* for 5 min at 4°C. The supernatant was transferred to a new 2.0 ml tube which was added 0.3 ml of Chloroform/isoamyl alcohol (24:1) per 1.5 ml of Trizol reagent. After the mix was centrifuged at 12000 × *g* for 10 min at 4°C, the aqueous phase was transferred to a new 1.5 ml tube in which an equal volume of supernatant of isopropyl alcohol was added. The mix was centrifuged at 12000 × *g* for 20 min at 4°C and then removed the supernatant. After washed with 1 ml 75% ethanol, the RNA pellet was air-dried in the biosafety cabinet and then dissolved by add 25∼100 μL of DEPC-treated water. Subsequently, total RNA was qualified and quantified using a NanoDrop and Agilent 2100 Bioanalyzer (Thermo Fisher Scientific, MA, United States).

Approximately 1 μg total RNA per sample was treated with Ribo-Zero^TM^ Magnetic Kit (Epicentre) to deplete rRNA. The retrieved RNA was fragmented by adding First Strand Master Mix (Invitrogen). First-strand cDNA was generated using random primers reverse transcription, followed by a second-strand cDNA synthesis. The synthesized cDNA was subjected to end-repair and then was 3′ adenylated. Adapters were ligated to the ends of these 3′ adenylated cDNA fragments. Several rounds of PCR amplification with PCR Primer Cocktail and PCR Master Mix are performed to enrich the cDNA fragments. Then the PCR products are purified with Ampure XP Beads. The final library was quality and quantitated in two methods: check the distribution of the fragments size using the Agilent 2100 Bioanalyzer, and quantify the library using real-time quantitative PCR (QPCR) (TaqMan Probe). The Qualified libraries were sequenced pair end on the Hiseq 4000 or Hiseq X-ten platform (BGI-Shenzhen, China).

### Proteomic Analysis

A 5 mm magnetic bead and an appropriate amount of Lysis Buffer 3 were added into a 1.5 ml tube containing the placenta sample. PMSF (final concentration: 1 mM) and EDTA (final concentration: 2 mM) were also added into the tube. The tube was then vortexed. After let stand for 5 min, DTT (final concentration: 10 mM) was added into the tube. The mix was shaken in a tissue grinder for 2 min (power = 50 HZ, Time = 120 s). After centrifuged at 25000 × *g* for 20 min at 4°C, the supernatant was transferred to a new tube which was added DTT (final concentration: 10 mM) again at 56°C water bath for 1 h. After returning to room temperature, IAM (final concentration: 55 mM) was added in a dark room and let stand for 45 min. Then cold acetone was added and let stand at −20°C for 2 h. This step was repeated until the supernatant was colorless. After centrifuged at 25000 × *g* for 20 min, a 5 mm magnetic bead and an appropriate amount of Lysis Buffer 3 were added into the precipitate. The mix was shaken in a tissue grinder for 2 min (power = 50 HZ, Time = 120 s). Finally the mix was centrifuged at 25000 × *g* for 20 min at 4°C, and the supernatant was taken for quantification. Bradford quantitative and SDS-PAGE were used for quality control of protein extraction.

Totally 2.5 μg trypsin enzyme was added into 100 μg protein solution for each sample (protein: enzyme = 40: 1) at 37°C for 4 h. Then the trypsin was added again according to the above ratio at 37°C for 8 h. Peptides after enzymolysis were desalted using Strata X column and vacuum dried. High pH reversed-phase separation was carried out by LC-20AB liquid system (Shimadzu). Peptides were measured by Liquid chromatography-MS/MS (LC-MS/MS) on an UltiMate 3000 UHPLC (Thermo Fisher Scientific). Q-Exactive HF (Thermo Fisher Scientific) was used for data-dependent acquisition (DDA) mode detection and data-independent acquisition (DIA) mode detection. The obtained DDA data were identified using the integrated Andromeda engine of MaxQuant (Cox and Mann, 2008) 1.5.3.30 (false discovery rate, FDR ≤ 1%). According to the results, Spectronaut (Bruderer et al., 2015) was used to construct a spectral library, of which the information was used to complete the deconvolution and extraction of DIA data. Quality control was carried out by mProphet algorithm. Finally significant quantitative results were obtained based on the Target-decoy model applicable to SWATH-MS (FDR ≤ 1%).

### Bioinformatics Analysis

The R package “edgeR” was used to identify differentially expressed messenger RNAs (mRNAs) and proteins in the samples. String (version 11.0) (Szklarczyk et al., 2019) and Metascape (Zhou et al., 2019) were used to assess the function of the differentially expressed mRNAs and proteins according to the gene ontology (GO) (Ashburner et al., 2000), the Kyoto Encyclopedia of Genes and Genomes (KEGG) (Kanehisa and Goto, 2000), and the Reactome pathway database (Jassal et al., 2020). Gene-sets enrichment analysis (GSEA) (Mootha et al., 2003; Subramanian et al., 2005) was used to analyze the KEGG pathways significantly correlated with concordant and discordant mRNA-protein expressions. String (version 11.0), Metascape and Cytoscape (Shannon et al., 2003) were used to construct visual interaction networks of differentially expressed mRNAs and proteins. Soapfuse (version 1.18) (Jia et al., 2013) was used to detect the fusion gene in each sample. The Genome Analysis Toolkit (GATK, version 3.4-0) (McKenna et al., 2010) was used to detect single nucleotide polymorphisms (SNP) information in each sample, which was further annotated by Snpeff (Cingolani et al., 2012). ASprofile^2^ was used to quantitatively detect splicing events of each sample. The data were analyzed in SPSS (version 25.0, IBM) and R (version 3.6.2).

## Results

A total of 10 placenta tissues (5 PAS patients: PAS1, PAS2, PAS3, PAS4, PAS5; 5 non-PAS patients: NPAS1, NPAS2, NPAS3, NPAS4, NPAS5) were sequenced for transcriptomic and proteomic analysis. There were no significant differences between PAS group and non-PAS group in maternal age and gestational age at delivery (*p* > 0.05, see Table 1 for detailed information of pregnant women).

**TABLE 1:** Detailed information of participants.

Patient ID	Age	Height (centimeter, cm)	Weight before delivery (kilogram, kg)	Previous caesarian section times	Type of placenta previa	Intraoperative blood transfusion	Gestational age at delivery (week)	Type of placenta accreta spectrum
PAS1	36	167	81	1	Complete	No	35.00	Increta
PAS2	28	160	64	1	Complete	Yes	36.56	Increta
PAS3	24	160	80	1	Complete	Yes	36.00	Increta
PAS4	37	160	80	1	Complete	Yes	36.00	Increta
PAS5	28	162	71.5	1	Complete	No	36.42	Increta
NPAS1	40	168	55	1	Complete	No	37.56	No
NPAS2	36	168	90	1	Complete	No	37.28	No
NPAS3	28	165	77	1	Complete	No	35.42	No
NPAS4	36	160	80	1	Complete	No	37.14	No
NPAS5	33	168	70	1	Complete	No	36.28	No

### Transcriptomic Profiling of Placenta Tissue

**FIGURE 1:**
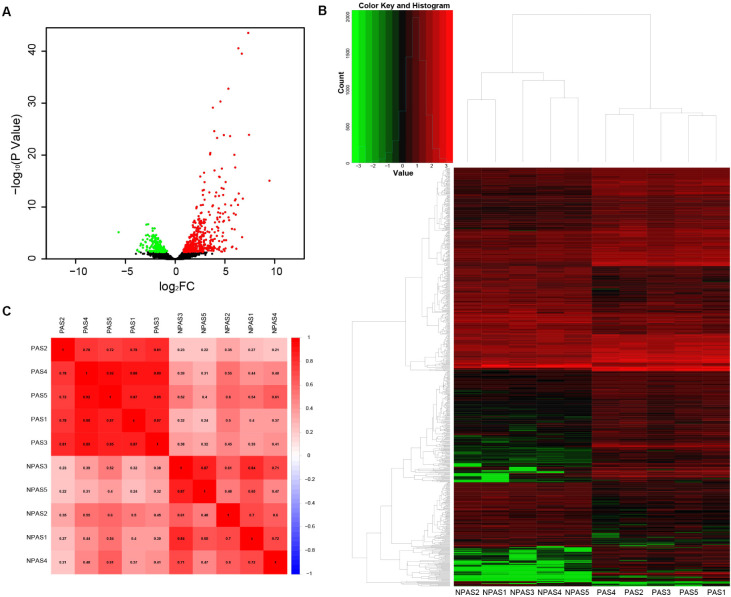
Analysis of differentially expressed mRNAs. **(A)** Volcano plot, **(B)** heat map, and **(C)** correlation between PAS group and non-PAS group.

**FIGURE 2:**
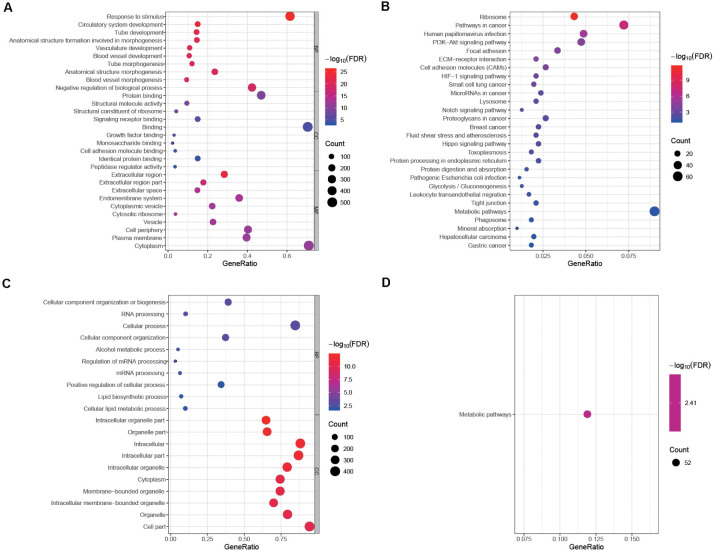
Enrichment analyses of differentially expressed mRNAs and DEPs. The top 10 biological process GO terms, cellular component GO terms, molecular function GO terms of **(A)** differentially expressed mRNAs and **(B)** DEPs sorted by false discovery rate (FDR) value. Significantly enriched KEGG pathways of **(C)** differentially expressed mRNAs and **(D)** DEPs. Some functions and pathways related to cell proliferation, adhesion and angiogenesis are not shown in this figure because the corresponding FDR values are not in the top 10. No molecular function GO term was enriched in the GO analysis of DEPs, so this part is not shown in **(B)**.

**FIGURE 3:**
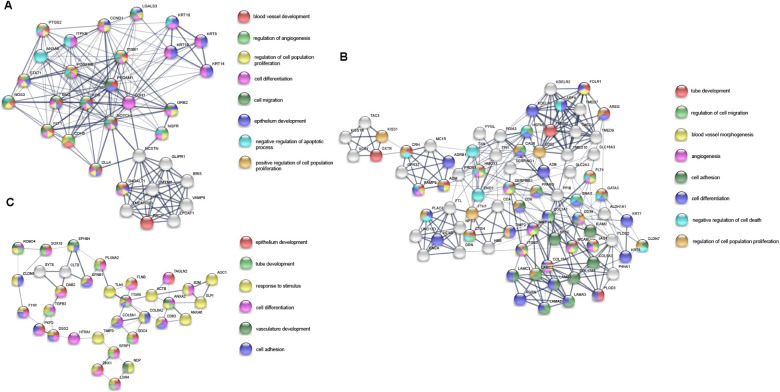
Three main gene clusters **(A–C)** related to cell proliferation, differentiation and vascular development in PPI network of differentially expressed mRNAs. The color of the nodes represents the participating function. The thickness of the edges indicates the strength of interaction evidence.

A total of 17,860 known mRNAs in placenta tissue were detected quantitatively. And 728 differentially expressed mRNAs (false discovery rate, FDR < 0.05, fold change >1.5) were obtained, including 481 up-regulated genes and 247 down-regulated genes (Figures 1A,B). Intra-group correlation was good in both PAS group and non-PAS group (Figure 1C). GO enrichment analysis in String showed that the differentially expressed mRNAs were mainly related to response to stimulus, vascular development and protein binding (Figure 2A). In addition, the biological processes related to cell proliferation and differentiation were also enriched (see Supplementary Table S1 for detailed results of GO analysis). KEGG enrichment analysis showed that differently expressed mRNAs were related to cancer and cell adhesion. PI3K/AKT signaling pathway, HIF-1 signaling pathway, notch signaling pathway and other classical pathways were enriched (Figure 2B, see Supplementary Table S2 for detailed results of KEGG analysis). In order to further explore the functional interaction of differentially expressed mRNAs, we constructed the interaction network with String, and isolated the tightly interacting gene clusters through MCODE in Cytoscape. Totally 26 gene clusters were obtained and three main clusters that were related to cell proliferation, differentiation and vascular development were shown in Figure 3.

### Proteomic Profiling of Placenta Tissue

**FIGURE 4:**
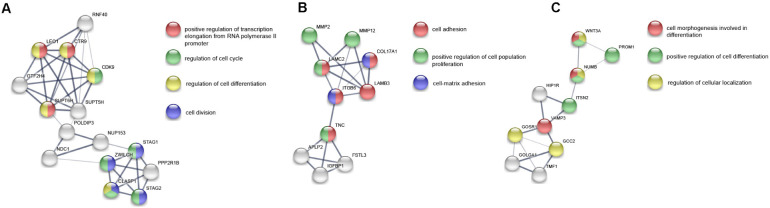
Three main gene clusters **(A–C)** related to cell proliferation, differentiation and vascular development in PPI network of DEPs. The color of the nodes represents the participating function. The thickness of the edges indicates the strength of interaction evidence.

A total of 4,800 known proteins were detected quantitatively. In the same way, we obtained 439 differentially expressed proteins (DEPs) (FDR < 0.05, fold change >1.5). The GO enrichment analysis showed that DEPs were mainly associated with cellular component organization or biogenesis and intracellular organelle (Figure 2C). The biological processes related to cell migration, differentiation and tube development were also enriched (see Supplementary Table S3 for detailed results of GO analysis). In KEGG enrichment analysis, DEPs were only associated with metabolic pathways (Figure 2D, see Supplementary Table S4 for detailed results of KEGG analysis). Similar to the differentially expressed genes, all the DEPs were constructed into a visual interaction network, and 20 gene clusters were obtained by MCODE analysis. The three main clusters related to cell proliferation, differentiation and vascular development were shown in Figure 4.

### Integrated Analysis of Transcriptome and Proteome

#### Overall Correlation and Functional Enrichment

**FIGURE 5:**
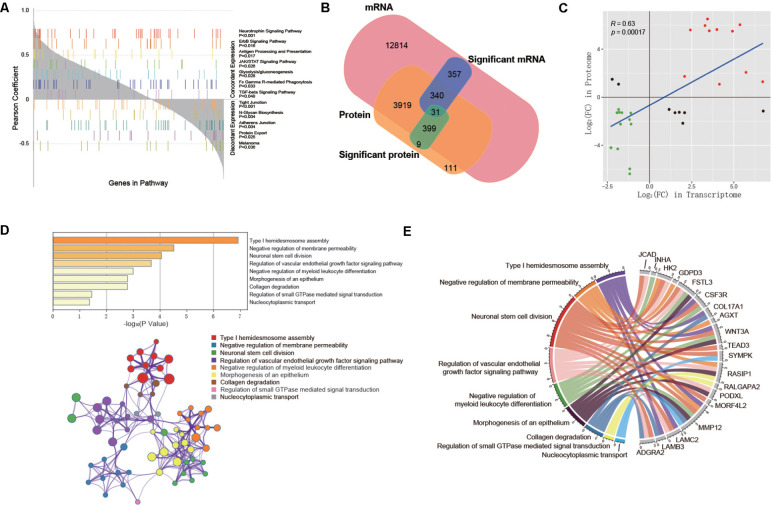
Integrative analysis between transcriptome and proteome. **(A)** KEGG pathways significantly correlated with concordant or discordant mRNA-protein expressions. Genes are aligned along the x axis by the rank of their Pearson correlation coefficient between mRNA and protein expression levels. Each color represents one significantly associated pathway, and each bar represents one gene in the pathway. **(B)** Venn diagram showing all identified mRNAs and proteins and their overlap. **(C)** The expression levels of 31 co-differentially expressed genes in the transcriptome and proteome. **(D)** Functional enrichment analysis based on Metascape. Bar graph demonstrates *P*-value of enriched clusters. Each node in network represents an enriched term of corresponding clusters. For large clusters, the network only displays the top 10 terms with *P*-value. The edge reflects the connection of functions and pathways. The size of the nodes reflects the number of genes, and the thickness of the edges reflects correlation level of terms **(E)** Chord diagram shows the hub genes with their representative enriched clusters.

First, we evaluated the correlation between mRNA expression and protein abundance in 10 samples. Specifically, we aggregated genes that were quantitatively detected in both the transcriptome and proteome and calculated the Pearson correlation coefficient between mRNA and protein expression levels of each gene in 10 samples. And 62.3% of the genes showed positive correlations (Supplementary Figure S1). Furthermore, GSEA was performed with the Pearson correlation coefficient as the rank of each gene in order to investigated whether the trend of mRNA-protein correlation was associated with specific KEGG pathways. As a result, we identified several pathways involved in cell proliferation, cell adhesion, vascular development, immunity, and metabolism (Figure 5A), which were all related to PAS.

#### Identification of Hub Genes and Related Functions

Subsequently, in order to explore the hub genes in PAS, we took the intersection of differentially expressed mRNAs and DEPs, and obtained a total of 31 co-differentially expressed genes (Figure 5B), among which the expression trend of 23 genes was consistent in transcriptome and proteome (Table 2). Pearson correlation analysis showed that the expression levels of these 31 genes in the transcriptome and proteome had a strong positive correlation (*R* = 0.63, *p* < 0.001, Figure 5C). Given the small sample size, we also ran a stochastic simulation to verify the reliability of these genes. In the simulation, 728 fake differentially expressed mRNAs and 439 fake DEPs were randomly selected from 17,860 mRNAs and 4,800 proteins, respectively, and the number of genes shared by both selected mRNAs and proteins was counted. Based on R (version 3.6.2), we performed the simulation 10,000 times and found that there was only a probability of 0.0019 that the number of shared genes was greater than or equal to 31, which further illustrated the reliability of the results.

**TABLE 2:** Detailed expression information of hub genes in transcriptome and proteome.

Hub genes	log_2_(FC) in transcriptome	FDR in transcriptome	log_2_(FC) in proteome	FDR in proteome
COL17A1	6.693	<0.001	1.288	0.003
MMP12	5.701	<0.001	2.064	<0.001
FSTL3	5.342	<0.001	6.037	<0.001
AGXT	4.899	<0.001	5.501	<0.001
INHA	4.123	<0.001	1.094	0.035
LAMC2	3.975	<0.001	5.614	<0.001
HK2	3.525	<0.001	5.534	<0.001
LAMB3	3.423	0.005	6.519	<0.001
APOBR	3.275	0.003	5.973	<0.001
MORF4L2	2.403	<0.001	5.581	<0.001
GDPD3	2.055	0.001	1.739	0.006
HID1	–2.308	<0.001	–4.205	0.012
CSF3R	–1.949	0.003	–1.287	0.001
WNT3A	–1.904	0.021	–4.3	0.007
RASIP1	–1.856	0.017	–1.034	0.030
PODXL	–1.819	0.049	–1.273	0.001
ADGRA2	–1.741	0.001	–2.249	<0.001
RALGAPA2	–1.662	0.031	–1.303	0.002
ERVW-1	–1.206	0.033	–1.87	<0.001
JCAD	–1.196	0.028	–6.379	<0.001
SYMPK	–1.19	0.011	–5.956	<0.001
TEAD3	–1.131	0.008	–1.326	0.001
INTS3	–1.116	0.023	–2.236	<0.001

*FC, fold change; FDR, false discovery rate.*

The functional enrichment analysis of the 23 hub genes with consistent expression trends was then carried out by Metascape, in which 9 function clusters including functions and pathways related to cell proliferation, adhesion and vascular development were enriched (Supplementary Table S5). The connection between each hub gene and 9 clusters is shown in Figures 5D,E. This was highly consistent with the above enrichment results in String and GSEA, which suggested that these 23 hub genes were likely to play an important role in the pathogenesis of PAS.

#### Regulation and Structure Variation of Hub Genes

**FIGURE 6:**
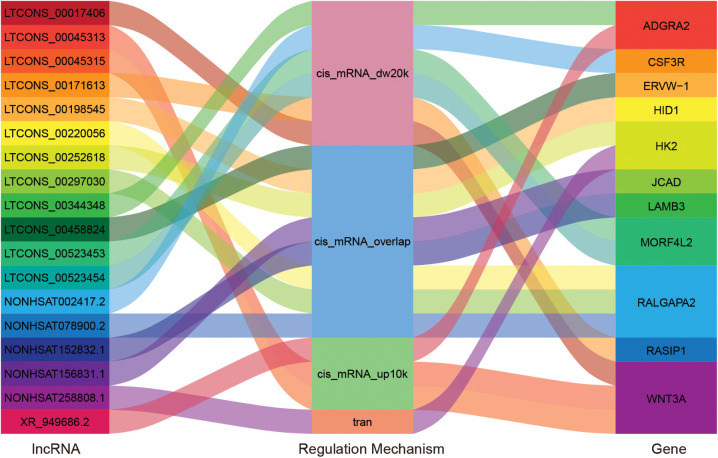
“Sankey” diagram for the correlation between lncRNA and hub genes. The middle column reflects regulation mechanism. cis_mRNA_up10k: Cis action, in which lncRNA is within 10 k upstream of mRNA; cis_mRNA_dw20k: Cis action, in which lncRNA is within 20 k downstream of mRNA; cis_mRNA_overlap: Cis action, in which lncRNA overlaps with mRNA; tran: Tran action.

**FIGURE 7:**
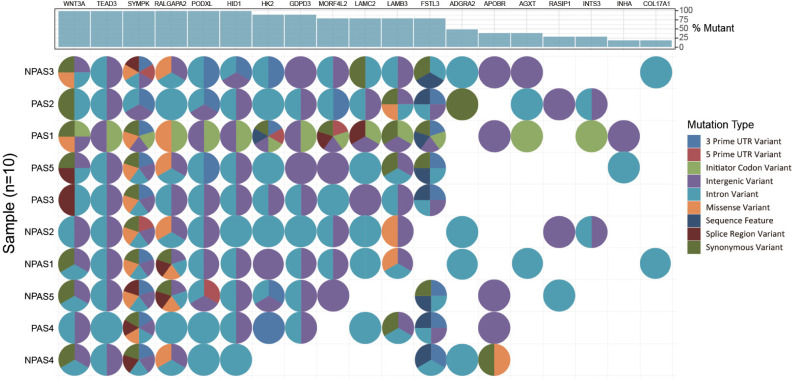
Waterfall plot demonstrates the types of SNP in hub genes.

**FIGURE 8:**
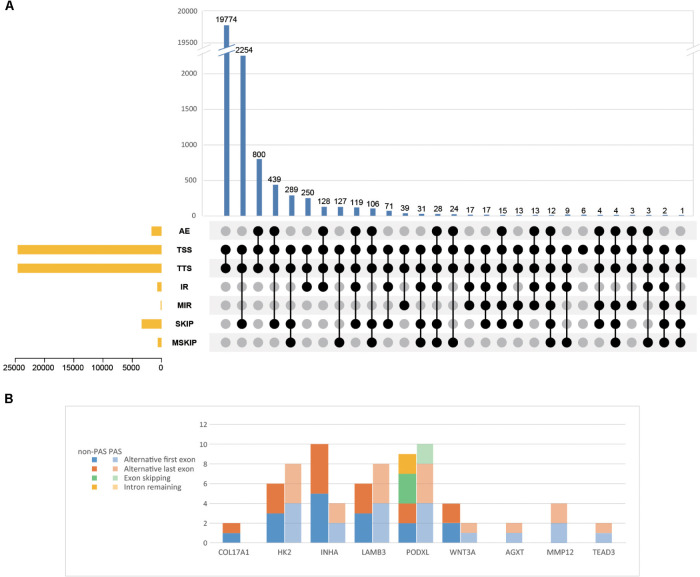
Alternative splicing events of **(A)** all identified genes and **(B)** hub genes. AE, alternative exon ends; TSS, alternative 5′ first exon; TTS, alternative 3′ last exon; IR, intron retention; MIR, multi-IR; SKIP, skipped exon; MSKIP, multi-exon SKIP.

**FIGURE 9:**
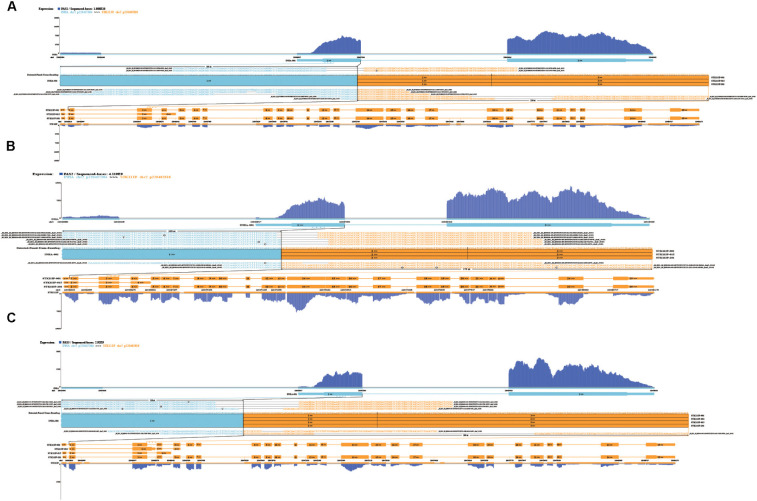
Gene fusion between INHA and STK11IP in **(A)** PAS1, **(B)** PAS2, and **(C)** PAS5.

In order to explore which long non-coding RNA (lncRNA) might regulate the expression of these 23 hub genes, we extracted the sequence results of lncRNA from placenta tissue and predicted the target genes. Totally 18 lncRNAs (including known and novel lncRNAs) might be involved in the regulation of some hub genes (Figure 6). The sequence of novel lncRNAs were shown in Supplementary Table S6. Pearson and Spearman correlation coefficients between lncRNA and mRNA were shown in Supplementary Table S7. In addition, we performed a series of analyses of hub genes in gene structure level, including SNP, alternative splicing and gene fusion. In terms of SNP, the presence of one or more types of SNP was found in most hub genes (Figure 7). The intron variation of *ADGRA2* (4 of 5 cases in NPAS group, *p* = 0.048, Fisher’s exact test, same for the following), the intergenic variation of *FSTL3* (4 of 5 cases in PAS group, *p* = 0.048) and the synonymous variation of *LAMB3* (4 of 5 cases in PAS group, *p* = 0.048) were significantly correlated with PAS. In the detection of alternative splicing events for all genes, 7 types of alternative splicing events, mainly alternative 5′ first exon (TSS) and alternative 3′ last exon (TTS), were found (Figure 8A). Four types of alternative splicing events were detected in the hub genes, among which the alternative splicing of *AGXT*, *MMP12*, and *TEAD3* were only detected in the PAS group (Figure 8B). The expression of the hub genes in alternative splicing events was analyzed by using the Mann-Whitney *U* test. The results showed that TSS of *LAMB3* at chromosome 1 sites 209652369 to 209652475 (*p* = 0.032) and TTS of *HK2* at chromosome 2 sites 74890797 to 74893354 (*p* = 0.032) were significantly associated with PAS. Finally, there was a gene fusion between *INHA* and *STK11IP* on chromosome 2, which was detected in PAS1, PAS2, and PAS5 (Figure 9).

## Discussion

In this study, we sequenced the placenta tissues of 5 women with PAS and 5 healthy pregnant women. And the results were analyzed systematically. Totally 23 hub genes which are closely related to PAS were screened out. As expected, most of these genes are directly related to cell proliferation and migration, as well as vascular development. There are also some hub genes related to trophoblast fusion, energy metabolism and DNA repair. Further, we found lncRNAs that might regulate the expression of hub genes and analyzed the hub genes at the gene structure level.

Totally 17,860 mRNAs and 4,800 proteins were detected quantitatively in this study. Although there was a strong positive correlation between the transcriptome and the proteome, only 23 genes were differentially expressed at both mRNA and protein levels and the expression trends were the same. This suggested that these genes might play a key role in PAS. And on the other hand, it also reflected the importance of post-transcriptional regulation in gene expression. The asynchronous expression of mRNAs and corresponding proteins has been reported in many studies (Dai et al., 2018; Wang D. et al., 2019). Therefore integrated analysis of transcriptomics and proteomics is of great benefit to the discovery of hub genes and underlying mechanisms (Maier et al., 2009).

Excessive invasion of extravillous trophoblasts (EVTs) is essential in the development of PAS. At this point, EVTs have a strong ability of proliferation and migration (Matsukawa et al., 2019). The functions and pathways related to cell proliferation and migration (cell adhesion) were enriched in all functional enrichment analyses in this study. Among the up-regulated hub genes, *MMP12* has been reported to be an important molecule in the process of trophoblast invasion and vascular remodeling under hypoxia (Chakraborty et al., 2016), and *FSTL3* can promote trophoblast proliferation, invasion, and lipid storage and inhibit trophoblast apoptosis (Xie et al., 2018). The functional enrichment analysis indicated that *GDPD3*, *LAMB3*, and *LAMC2* were involved in the PI3K/AKT signaling pathway to promote cell proliferation and migration, which was confirmed in the related reports (Rao et al., 2017; Wang Q. et al., 2019; Zhang H. et al., 2019). *MORF4L2* and *COL17A1* could also promote cell proliferation and migration (Shadeo et al., 2008; Thangavelu et al., 2016). In addition, up-regulated expression of *HK2* can inhibit apoptosis (Xu et al., 2017). Among the down-regulated hub genes, *TEAD3* was reported to be down-regulated during trophoblast implantation into the endometrium (Bai et al., 2018); *HID1*, *WNT3A*, and *SYMPK* were also associated with cell proliferation, invasion and adhesion (Sonderegger et al., 2010; Chang et al., 2012; Aydin and Arga, 2019). In general, these genes played a role in reducing cell adhesion, promoting cell proliferation and migration, and inhibiting cell apoptosis, which is very important in the EVTs invasion into the myometrium. It should also be noted that both *PODXL* and *CSF3R* have been reported to promote cell proliferation or migration (Yeo et al., 2018; Zhang J. et al., 2019), but their expression in the PAS group was down-regulated in this study. Co-expression of enhancement signals and suppression signals in maternal regulation of placental cells were found in order to maintain the homeostasis (Pavlicev et al., 2017). Increased proliferation and migration of EVTs might trigger some negative feedback mechanism that led to low expression of *PODXL* and *CSF3R*.

Another pathological change accompanied by EVTs invasion in PAS is the remodeling of maternal blood vessels (Tantbirojn et al., 2008). The functional enrichment analysis in various parts of this study also enriched multiple functions and pathways related to vascular development, including the PI3K/AKT signaling pathway that was reported to be related to PAS (Long et al., 2020). The hub genes related to vascular development included *HK2*, *RASIP1*, *ADGRA2*, and *JCAD*. It has been reported that HK2 promoted aerobic glycolysis and activated p38-MAPK signal conduction in angiogenesis of melanoma (Lu et al., 2019). *HK2* was highly expressed in the PAS group and might be involved in maternal vascular remodeling. The remaining three genes, including *RASIP1*, *ADGRA2*, and *JCAD* were low expressed in the PAS group. *RASIP1* increased the stability of endothelium connection (Koo et al., 2016), which was not conducive to vascular remodeling; *ADGRA2* and *JCAD* were also found to be related to the inhibition of vascular development (Vallon et al., 2010; Hara et al., 2017). These molecules may act synergistically to promote the formation of blood vessels.

It is worth mentioning that some other hub genes and functions might also be involved in the pathogenesis of PAS. *ERVW-1* and *INHA* were the hub genes in this study. It has been reported that *ERVW-1* induced the fusion of trophoblast cells and reduced the proliferation and migration ability of trophoblast cells (Dunk et al., 2018), while another study showed that the expression level of *INHA* in cytotrophoblast cells was higher than that in syncytiotrophoblast cells (Azar et al., 2018). We found that *INHA* was up-regulated and *ERVW-1* was down-regulated in the PAS group. Therefore, we thought that the fusion of trophoblast cells was probably inhibited by enhanced expression of *INHA* and inhibited expression of *ERVW-1*, thereby ensuring the strong proliferation and migration ability of trophoblast cells. In the KEGG pathways significantly correlated with concordant and discordant mRNA-protein expressions, the glycolysis/gluconeogenesis pathway was found to be significantly associated with PAS. *HK2*, one of the hub genes involved in this pathway, was highly expressed in the PAS group. *HK2* depletion was shown to inhibit glycolysis in hepatocellular carcinoma and increased cell death (Xu et al., 2017). Similarly, the high expression of *HK2* in the PAS group might promote glycolysis and maintain energy supply during EVTs invasion and vascular remodeling. Among the hub genes, *APOBR* and *AGXT* are, respectively, related to lipid metabolism and alanine metabolism (Brown et al., 2000; Montioli et al., 2012), both of which were up-regulated in the PAS group and might also participate in the energy metabolism process in PAS. In terms of immunity, dNK cells was reported to regulate the invasion ability of EVTs by secreting colony-stimulating factors (CSFs) (Vento-Tormo et al., 2018). *CSF3R* expression was low in the PAS group, which might be related to the regulation of EVTs by dNK cells. In addition, *INTS3*, a gene widely involved in DNA repair (Yang et al., 2015), was found to be downregulated in the PAS group, which might cause mutations in some genes and RNA splicing (Yoshimi et al., 2019). We speculated that the SNP and alternative splicing events, which were found in this study and significantly related to PAS, might be related to the low expression of INTS3.

In this study, multiple SNP and alternative splicing events of hub genes were found. Intron variation of *ADGRA2*, intergene variation of *FSTL3* and synonymous variation of *LAMB3* were significantly related to PAS (*p* < 0.05). SNP of *ADGRA2* was reported to be related to angiogenesis of embryonic central nervous system (Weinsheimer et al., 2012), while SNP of *FSTL3* and *LAMB3* have not been reported. However, both SNP and alternative splicing events of *LAMB3* were significantly related to PAS in this study. In some diseases, synonymous mutations can affect splicing events and cause phenotypic changes (Liu et al., 2018; Sharma et al., 2019). It is worth exploring whether there is a similar mechanism for *LAMB3* in PAS. In addition, the alternative splicing of *HK2* was also significantly related to PAS. It has been reported that *HK2* can be amplified into two different fragments through alternative splicing and influence the invasion and metastasis of prostate cancer (Slawin et al., 2000). Besides, we also found the gene fusion between *INHA* and *STK11IP*. Gene fusion is one of the main causes of tumor formation, which is often used as a tumor diagnosis and prognostic marker (Mertens et al., 2015). The gene fusion between *INHA* and *STK11IP* only occurred in 3 of 5 PAS placenta tissues, so it might not be used as an accurate genetic marker of PAS, but whether it has the potential to assist diagnosis remains to be studied. Similarly, although various SNP and alternative splicing events were found to be significantly related to PAS, considering the small sample size in this study, more samples with sequencing data are needed to verify these variations.

Although some PAS-related molecules and mechanisms have been reported in other studies, this is the first time that the integrated analysis of transcriptomics and proteomics has been performed to systematically identify hub genes and gene structure variations involved in the pathological process of PAS. In addition, lncRNAs that may regulate hub genes were identified and analysis at the gene structure level was carried out, which is also one of the advantages of this study. Meanwhile, this study also has some shortcomings. First, the sample size of the study is small, and it is susceptible to external factors. In order to make up for this deficiency, the patients enrolled in this study were limited to having a history of cesarean section and no other history of uterine cavity operation or uterine-related diseases, which excluded the interference of different PAS etiologies and some other potential confounding factors. However, the hub genes and related functions discovered in this study still need to be verified by a large number of further experiments. Second, there were few omics studies related to PAS. Only one relevant sequencing data set can be found on Gene Expression Omnibus (GSE126552), in which PAS and normal placenta tissue was sequenced to find potential biomarkers and molecular mechanism of PAS. We downloaded and analyzed the data set, but unfortunately, there were no differentially expressed lncRNAs and mRNAs in this data set. Therefore, the hub genes found in this study cannot be effectively verified externally. Nevertheless, several hub genes, including *MMP12*, *FSTL3*, *TEAD3*, *ERVW-1*, and *INHA*, have been confirmed in other studies to regulate the invasion of EVTs or vascular development, which has been mentioned in the corresponding part of the discussion.

Omics analysis provides a new method for systematically exploring the hub genes of PAS. However, except for tumors, omics study of most other diseases is still in its infancy. This systematic study provided a genetic basis for further in-depth study on the underlying regulatory mechanism of PAS. With the progress of omics studies in PAS and the increasing sample size, the genetic features of PAS will be more clear and the structure variation of hub genes will be more statistically significant, which will contribute to the gene-level intervention in the development of PAS.

## Data Availability Statement

The RNA sequencing data was uploaded to NCBI Sequence Read Archive. The BioProject Number is PRJNA627183 and you can learn more about the data at http://www.ncbi.nlm.nih.gov/bioproject/627183.

## Ethics Statement

The studies involving human participants were reviewed and approved by the Medical Ethics Committee of China Medical University. The patients/participants provided their written informed consent to participate in this study.

## Author Contributions

CQ and BC designed the study. CQ supervised the whole study. BC and DW analyzed the data and wrote the manuscript. YB, JL, TY, and NL collected and sequenced the samples. All authors contributed to the article and approved the submitted version.

## Conflict of Interest

The authors declare that the research was conducted in the absence of any commercial or financial relationships that could be construed as a potential conflict of interest.

## Footnotes

1 http://www.who.int/classifications/icd

2 http://ccb.jhu.edu/software/ASprofile
