# Supplementary material for: Systematic Identification of Hub Genes in Placenta Accreta Spectrum Based on Integrated Transcriptomic and Proteomic Analysis
Source: Front Genet. 2020 Sep 15;11:551495. doi: 10.3389/fgene.2020.551495 (PMC7522549; doi:10.3389/fgene.2020.551495)
Supplement: Supplementary file 1 [file Data_Sheet_1.DOCX]

Supplementary Material

# Supplementary Figure S1


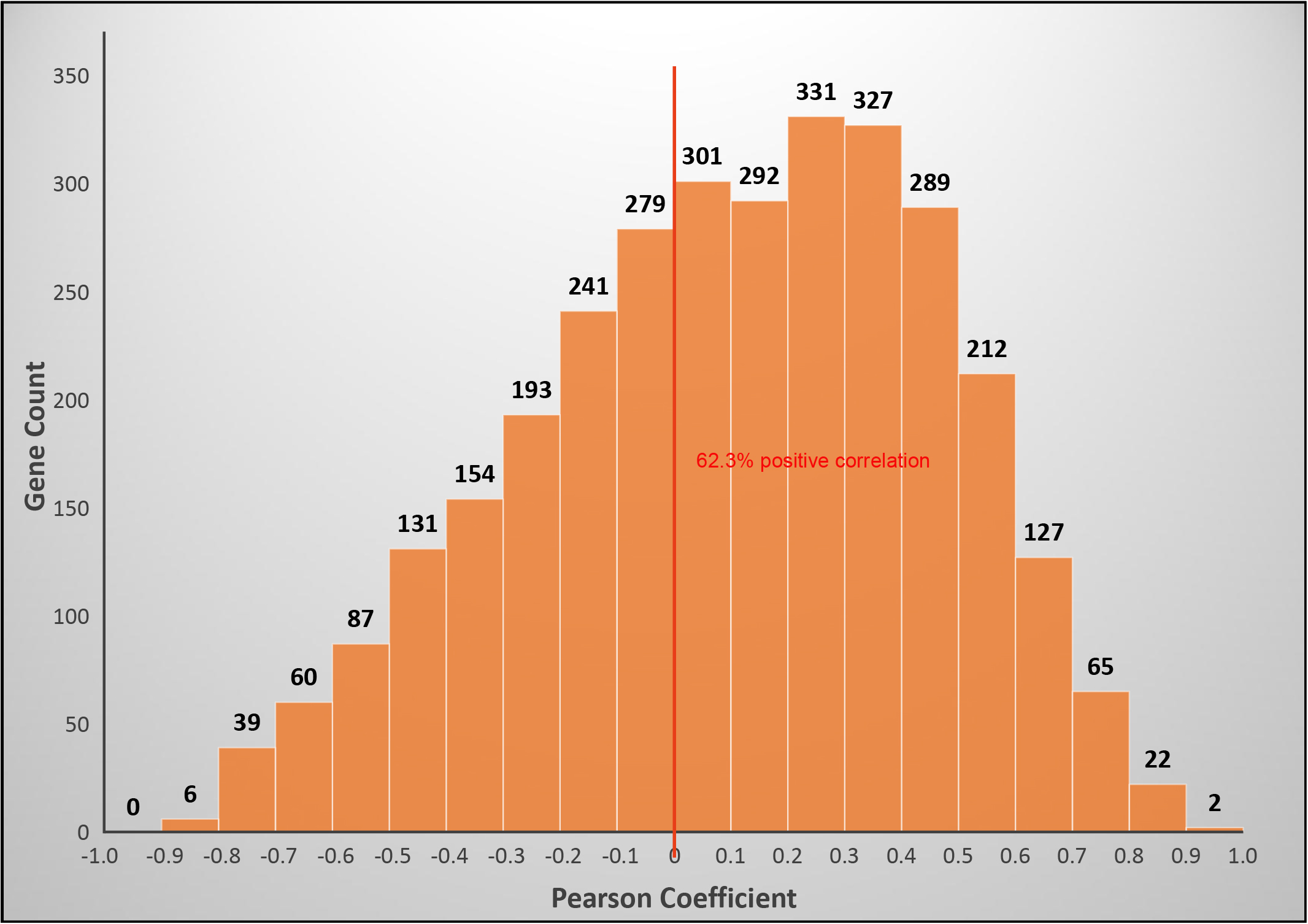


**Supplementary Figure 1.** Distribution of Pearson correlation coefficient between mRNA and protein expression levels. Totally 62.3% of the genes were positive correlated.
